# Divergent elbow dislocation with radial shaft fracture, distal ulnar deformation, and distal radioulnar joint instability: an unclassifiable Monteggia variant

**DOI:** 10.1007/s10195-013-0239-x

**Published:** 2013-04-07

**Authors:** Joseph L. Laratta, Richard S. Yoon, Matthew A. Frank, Kenneth Koury, Derek J. Donegan, Frank A. Liporace

**Affiliations:** 1 Division of Orthopaedic Trauma, Department of Orthopaedic Surgery, UMDNJ—New Jersey Medical School, Newark, NJ USA; 2 Division of Orthopaedic Trauma, Department of Orthopaedic Surgery, NYU Hospital for Joint Diseases, 301 E 17th Street, Suite 1402, New York, NY 10003 USA; 3 Division of Orthopaedic Trauma, Department of Orthopaedic Surgery, Hospital of the University of Pennsylvania, Philadelphia, PA USA

**Keywords:** Monteggia, Bado, Elbow dislocation, DRUJ, Radial shaft, Distal ulna, Both bones

## Abstract

Originally described by Monteggia and later classified by Bado, elbow dislocations with concurrent radial and ulnar shaft fractures with distal radioulnar joint (DRUJ) disruption are considered operative cases with high-energy injurious etiologies. Here, we present an unclassifiable Monteggia variant fracture suffered through a high axial load mechanism in a 47-year-old female. The fracture pattern initially exhibited included a divergent elbow dislocation, a radial shaft fracture, plastic deformation of the distal ulna, and DRUJ instability. Here we describe the pattern in detail, along with definitive treatment and clinical outcome at 1 year follow-up.

## Introduction

Originally described by Giovanni Monteggia in 1814, the Monteggia fracture pattern has gone through a few revisions [1]. Further subclassified by Bado and later by Jupiter, this fracture pattern warrants immediate attention and (usually) a trip to the operating room for definitive reconstruction [2–5]. In general, fracture involvement of both the radius and ulna with a concomitant radial head dislocation describes the fracture pattern [6]. In attempts to further understand the injury, other Monteggia fracture variants and equivalents have also been described [2, 7].

However, to our knowledge, no report has described the injury sustained in our patient: divergent elbow dislocation with concomitant radial shaft fracture, plastic deformation of the distal ulna shaft, and distal radioulnar joint (DRUJ) instability—a unique fracture pattern that has similar characteristics to the Monteggia lesion, but with obvious differences. Herein, we report the reconstruction and outcome of a novel Monteggia fracture variant in which the radial head and ulna dislocate in opposite directions—a contradistinction to the original observations that served as the basis for Bado’s classification scheme.

## Case report

Informed consent was obtained from the patient prior to starting this manuscript.

A 47-year-old female presented to the outpatient office complaining of right elbow pain after falling down two flights of stairs onto an outstretched hand while intoxicated, 10 days prior to presentation. Initial treatment at an outside institution included attempted closed reduction and posterior splint placement. Upon examination, she was found to have limited range of motion (ROM), intact skin, motor, and neurologic function, and palpable distal pulses in the right upper extremity. Furthermore, she also had limited ROM of the right wrist with laxity to DRUJ shuck as compared to the contralateral limb.

**Fig. 1:**
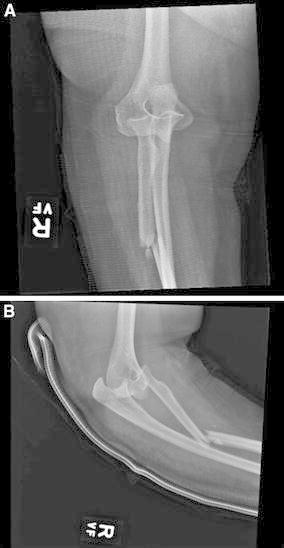
AP (**a**) and lateral (**b**) radiographs of the right elbow reveal a fracture of the radial shaft with the apex dorsal. The proximal radius is dislocated anteriorly and the ulna posteriorly with respect to the humerus

**Fig. 2:**
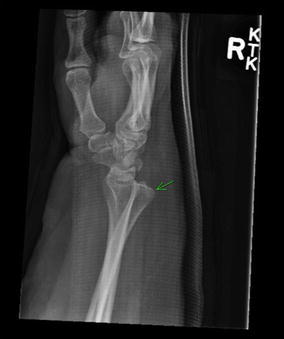
Lateral radiographs of the *right wrist* demonstrating distal radioulnar joint disruption and dorsal ulnar dislocation (*green arrow*)

**Fig. 3:**
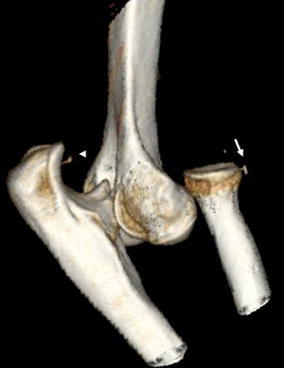
Lateral 3D reconstruction of CT scans of the *right elbow* confirm the divergent dislocation. Note the small radial head fracture. The coronoid process is abutting the posterior cortex of the medial humeral condyle. A tiny bone fragment off the coronoid process is seen between the olecranon fossa and humerus (*arrowhead*)

Radiographs demonstrated a comminuted and displaced proximal third radial shaft fracture with associated proximal radioulnar, radiocapitellar, and ulnohumeral joint dislocations (Fig. 1a, b). Distally, DRUJ disruption was obvious both radiographically and clinically (Fig. 2). Advanced CT imaging revealed radial head and coronoid process fractures along with posterior dislocation of the ulna and anterior dislocation of the radius in relation to the humerus (Fig. 3).

The patient was taken to the operating room for definitive reconstruction. First, the proximal third radial shaft fracture was addressed via a volar Henry approach and a small fragment plate employing both non-locked and locked fixation. Next, dissection proceeded through the Kaplan interval. The comminuted fragments of the radial head and coronoid process were debrided. In an attempt to stimulate fibrocartilaginous synthesis, a 1.6 mm Kirschner wire was then used to drill approximately 1 cm^2^ of osteochondral loss on the radial head, the coronoid process, and the medial epicondyle of the distal humerus. A completely avulsed MCL was noted during ulnar nerve transposition and was repaired using a 3.5 mm suture anchor inserted into the distal-most aspect of the midsagittal plane of the medial epicondyle.

The stability of the elbow joint was assessed while maintaining manual tension of the LCL, with sutures ultimately incorporated into its definitive repair. The elbow was stable in flexion, varus and valgus, but unstable anteriorly when extended to less than 40°. To supplement the repair for 6 weeks, a unilateral hinged external fixator was placed. Next, the LCL was repaired utilizing the ex-fix guide pinhole as the isometric point for insertion of a 5.5 mm suture anchor. Following repair of the annular ligament, the elbow could be passively ranged in a stable fashion.

Next, attention was turned to the DRUJ. Shuck testing under fluoroscopy confirmed clinical findings. The joint was reduced in supination and stabilized with two parallel 2.0 K-wires to engage four cortices from the distal ulna to the distal radius. Finally, the external fixation device was then locked in 70° of flexion, and all wounds were closed primarily.

The patient tolerated the procedure well and was discharged on her second postoperative day. Her external fixator was locked at 70° of flexion for the first 2 weeks of follow-up. At the 2-week follow-up, her external fixation device was unlocked, allowing full range of motion, and occupational therapy was started. At the 3-week follow-up, her active ROM was 0–70°.

At the 5-week follow-up, the patient reported hyperextending her elbow a few days prior when inebriated and falling against a wall. Her elbow AROM was painless and improved to 0–90° with mild but improving wrist stiffness. Radiographs revealed a stable reduced DRUJ and proximal radioulnar joint (PRUJ), although there was some concern regarding the ulnohumeral articulation. CT scan confirmed a small posterior subluxation of the ulna on the humerus. She was then placed in a posterior splint at 90° of flexion, and she was taken to the operating room for ex-fix adjustment, manipulation under anesthesia, and removal of the DRUJ K-wires at 6 weeks postoperatively.

In the operating room, the elbow exhibited stable ROM from 0 to 130° and a concentrically reduced elbow was obtained via ex-fix adjustment. Owing to the patient’s previous slight posterior subluxation and concerns with compliance, the hinge was re-locked for one more week to allow further soft tissue healing. Hardware was removed from the DRUJ and shuck testing demonstrated a stable, well-reduced DRUJ. The patient tolerated the procedure well and was discharged the same day.

**Fig. 4:**
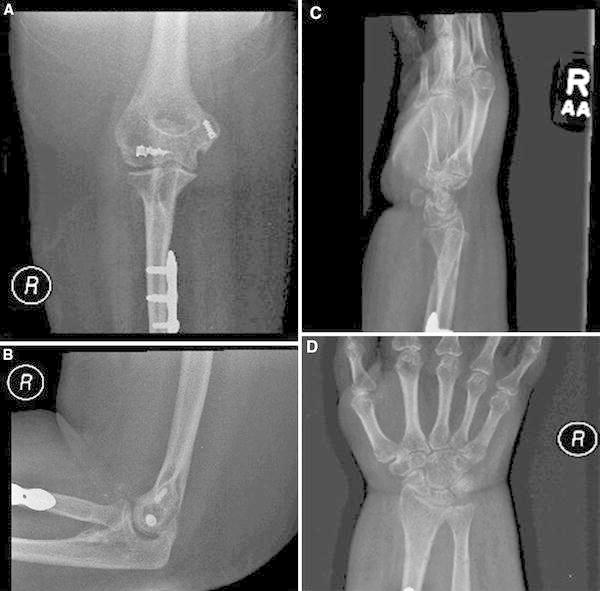
AP (**a**) and lateral (**b**) radiographs of the elbow exhibiting a well-healed and concentrically maintained elbow with a stable distal DRUJ (**c–d**)

**Fig. 5:**
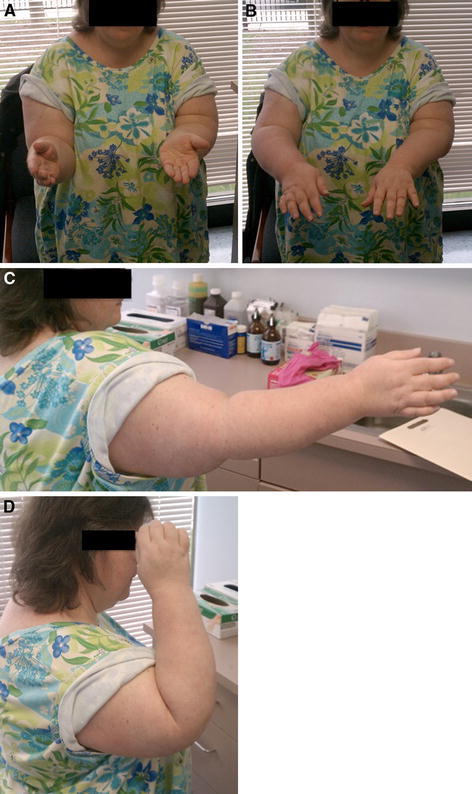
Photographs at the 1-year follow-up clinical examination demonstrating supination of 45° (**a**), pronation of 80° (**b**) extension of 5° (**c**), and flexion of 95 ° (**d**) in the affected extremity

At the 9-week follow-up, the patient noted increased ROM in the wrist as well as pain-free ROM of the elbow and shoulder. She remained neurovascularly intact. X-ray examination revealed a well-reduced DRUJ, PRUJ, and elbow joint with radial shaft union. At this time, the calculated DASH score was 38.8 (Fig. 4a, b). She remained in outpatient physical and occupational therapy for 15 weeks, and continued the exercises at home. At the 1-year follow-up, she had symmetric AROM flexion/extension of the elbow. She was able to supinate her forearm to approximately 45° and pronate it to approximately 80° (Fig. 5a–d). At the 1-year follow-up, the calculated DASH score had improved to 5.8.

## Discussion

Monteggia fractures are typically associated with high-energy impact—they are most commonly due to a fall on an outstretched hand in forced pronation, as was evidenced in our case. As ulnar displacement increases, the radial head is levered out of the joint, resulting in the classic deformity [8, 9]. Despite this, Monteggia fractures and their variants are rare and are often missed injuries.

Although rare, the Monteggia fracture has gained notoriety among the orthopedic community in light of its notoriously poor outcomes. The displacement by muscle forces across the fracture site and associated ligamentous injury has been shown to be a source of significant morbidity in the past, especially before the routine use of contoured compression plates for treatment [10, 11]. Bado type II and injuries with associated radial head and coronoid fractures have historically portended poor clinical outcomes [3]. Potential complications of Monteggia fractures include persistent dislocation of the radial head, elbow stiffness, forearm synostosis, nerve palsy, and residual forearm deformity [12]. There have been reports of persistent dislocation of the radial head caused by median and radial nerve entrapment, resulting in subsequent nerve palsy [13, 14].

Treatment of complicated Monteggia fractures and their variants remains a challenge. Much of the literature reporting on treatment and outcomes combines pediatric and adult injuries. Although closed treatment remains standard for most pediatric Monteggia fractures, internal fixation is mandatory in adults [15]. Despite the potentially disabling and often unpredictable complications inherent to open fixation, recent studies have shown satisfactory results in 55–83 % of Monteggia fractures when treated with anatomic reduction and stable plate fixation that permits early mobilization [4, 16]. Anatomic alignment of the ulnar fracture with restoration of the trochlear notch typically allows for a manageable reduction of the proximal radiocapitellar joint and, consequently, a satisfactory result.

Achieving a satisfactory result for our patient coincided with the aforementioned goals for reconstruction—anatomic and stable reconstruction of the PRUJ with definitive restoration of the forearm ring. Representing an unclassifiable Monteggia variant-type injury, our patient’s initial forecast did not bode well on first presentation. However, paying particular attention to maintaining concentric stability at the elbow along with early, protected ROM via plate fixation and combined hinged ex-fix utilization resulted in a good clinical outcome, despite the nonadherent efforts put forth by our patient [17]. While the overall use of microfracture remains controversial, it was utilized in our case to provide our patient with every chance of maintaining long-term painless, full elbow ROM and function. Long-term follow-up with possible re-exploration will be necessary to truly assess the role of microfracture in this setting.

In summary, combined plate fixation, ligamentous repair, DRUJ stabilization, and placement of a hinged external fixator proved to be an effective strategy to achieve union and restore functional status in our patient with a high-energy, unclassifiable Monteggia fracture variant. Paying particular attention to anatomic restoration of the forearm ring with concentric elbow stability is of paramount importance. However, this represents just one case in an already rarely occurring fracture pattern, and additional studies will be necessary to prove the true efficacy and reproducibility of this treatment strategy. Furthermore, the role of microfracture in this injury setting also needs to be further explored, as it may prove as a useful adjunct in maintaining fluid elbow function.
